# A Conceptual Model of Experiences With Digital Technologies in Aging in Place: Qualitative Systematic Review and Meta-synthesis

**DOI:** 10.2196/34872

**Published:** 2022-09-09

**Authors:** Mareike Hechinger, Diana Hentschel, Christine Aumer, Christian Rester

**Affiliations:** 1 Deggendorf Institute of Technology Faculty of Applied Healthcare Science Deggendorf Germany

**Keywords:** older adults, old age, assistive device, aging in place, home modification, independent living, telemedicine, assistive technology, ambient assisted living, assisted living, community living, chronic disease, chronic condition, chronic illness, elder, older adult, systematic review, meta-synthesis, digital technology, mobile phone

## Abstract

**Background:**

Older adults with chronic illnesses or dependency on care who strive to age in place need support and care depending on their illness. Digital technology has enabled the possibility of supporting older adults in their wishes to age in place. However, current studies have mainly focused on the solitary evaluation of individual technologies or on evaluating technologies for specific illnesses.

**Objective:**

This study aimed to synthesize research on the experiences of older people from the Western culture with chronic illnesses or care needs and their families with digital technology for aging in place. From the meta-synthesis, a model was derived that can be useful for the development of assistive devices in old age and that can support health care providers and professionals in their work with affected individuals.

**Methods:**

A systematic review and qualitative meta-synthesis was performed using an inductive approach, as proposed by Sandelowski and Barroso. We performed a systematic literature search in 6 databases from 2000 to 2019, with an update in 2021 and, in addition, conducted a hand search in 2 databases, relevant journals, and reference lists. The results of each study were analyzed using initial and axial coding, followed by theoretical coding. A conceptual model was derived.

**Results:**

A total of 7776 articles were identified. Articles were screened independently by 2 authors based on the eligibility criteria. Finally, of the 7776 studies, 18 (0.23%) were included in the meta-synthesis. The derived conceptual model describes older adults with chronic illnesses or dependency on care and their family members in an individual process of reflection and decision-making, starting with the use of a digital device. Older adults live in times of change. They experience stable and unstable times of illness as they are part of a changing digital world. Hence, older adults and their families consider digital technology a solution to their current situation. As they become familiar with a specific digital technology, they refine their needs and demands, gain confidence in its use, and note its advantages and disadvantages. They weigh hopes, needs, demands, and experiences in a process of reflection to decide on convenience and inconvenience. Independent of their decision, they achieve peace of mind either with or without digital technology. This process can restart repeatedly during the illness trajectory of older adults.

**Conclusions:**

This study promotes a differentiated understanding of older adults’ experiences with digital technology. The conceptual model can be useful for the development of assistive technology in old age. Moreover, it can guide health care professionals in their work with older adults and their families to provide individual counseling to find the appropriate digital technology for their respective situations.

## Introduction

### Background

Aging is closely linked to the question of how and where one wants to live in later life. Aging in place is a central wish for many older adults. They associate it with autonomy, continuity in daily life, privacy, and memories that give meaning to life [1,2]. The concept of *aging-in-place* can be viewed through 5 key themes. In addition to place, these are social networks, support, technology, and personal characteristics [3]. On the basis of these key themes, we defined aging in place as living in one’s own home for as long as possible while maintaining social networks and respecting older adults’ autonomy in deciding what professional or technical assistance is needed. Support, as part of the aging-in-place concept, refers to the likelihood of developing (further) health or care needs in the aging process. The World Health Organization defined healthy aging as “the process of developing and maintaining the functional ability that enables well-being in older age” [4]. Healthy aging at home is increasingly being supported by digital-technical solutions. The diversity of digitally assisted living technologies has grown significantly. Nilsson et al [5] reviewed digital technologies for older adults and their informal caregivers as interventions for healthy aging, using the corresponding World Health Organization framework. Interventions focused “on physical capacity and function, on managing the symptoms of dementia and cognitive impairment, on supporting functioning in daily life and on self-caring with a chronic disease” [5]. Interestingly, the ability to participate in society was not addressed at all, although it is a central aspect of aging in place.

For existing qualitative reviews or meta-syntheses of the experiences of older adults living at home and using digital technologies, the study situation is as follows. In a meta-synthesis, Larsen et al [6] analyzed the process of becoming a user of assistive technologies and identified facilitating factors. They had narrow criteria for technology inclusion and did not focus on linking aging in place with assistive technology. In a qualitative systematic review and meta-synthesis, Moore et al [7] specified whether users integrate devices into everyday life with the degree to which motivation, ease of use, and device purpose match. In a meta-ethnography, Rosenwohl-Mack et al [8] specified the concept of aging in place as a balance of threats, agency in relation to identity, connectedness, and place. Owing to the holistic view of aging in place, the connection between aging in place and the use of technological-digital tools from the user perspective was only marginally addressed.

The systematic review by Pol et al [9] on the use of digital technologies by older adults summarizes studies that mainly examine healthy volunteers without care needs and focus on technical aspects of sensory monitoring rather than on the applicability in the daily life of a person aging in place. In a scoping review, Rodrigues et al [10] examined the extent to which web-based interventions could address loneliness in the context of the COVID-19 pandemic. Both studies show little visibility of the subjective perspective in the use of digital technologies in the aging in place of older adults. Other studies have concentrated on a specific group, such as the study by Chen and Yeh [11] on individuals with diabetes. In a meta-synthesis on self-monitoring of diabetes, they showed the experiences of patients on 5 different topics that can help health care professionals to better communicate with patients. Research on this topic has often focused on benefits, barriers, or ambivalences. The scoping review by Raja et al [12] shows such ambivalences in the context of telehealth, making life easier and the opposite. The scoping review also shows that the absence and presence of social support facilitated the use of digital technologies. A systematic review by Stargatt et al [13] stated the benefits of digital storytelling for older people with dementia living in the community. The improvements were concerned with mood, memory, social engagement, and quality of relationships in older people with dementia.

A research gap that emerges is that the experience of older people living at home when using digital technologies is still included less in the general evidence. The perspectives of both older adults and family members should be addressed when developing technical solutions [5,14]. The experiences of older adults aging in place and their family members are valuable for the development of a conceptual model overarching the varying illnesses, levels of care needs, or tested digital technologies.

### Objective

Therefore, we intended to synthesize qualitative research to gain insights into the experiences of older adults and their family members. The following research question guided this study: what experiences do older adults with chronic illness or dependency on care and their family members have with digital technology referring to aging in place? The aim was to gather and synthesize qualitative research on the experiences of older adults and their family members in using digital technology to overcome challenges in aging, chronic illness, and the need for care in daily life. We intended to derive a conceptual model that illustrates the patterns identified within these experiences. As such, the model can be useful for the development of assistive digital technology in old age, as well as support health care providers and health care professionals in their work with specific populations. The term *healthcare professionals* refers to nurses, physicians, and therapists.

## Methods

### Overview

A qualitative systematic review and meta-synthesis was conducted. Conducting a meta-synthesis allows for the interpretive integration of several studies, resulting in findings based on a larger sample than would be possible in a single qualitative research study. It is a systematic and inductive approach in which the results of the included studies are interpreted as a whole. Consequently, coherent experiences of the research topic can be explained and described as they enable an understanding that goes beyond a mere summary of all findings. The approach by Sandelowski and Barroso [15] was used with the following recommended steps: (1) formulating a purpose, (2) searching and retrieving literature, (3) appraising findings, (4) classifying findings, (5) conducting a meta-summary, and (6) developing a meta-synthesis. To ensure methodical rigor, we followed the ENTREQ (Enhancing Transparency in Reporting the Synthesis of Qualitative Research) checklist [16].

### Searching for and Retrieving Relevant Studies

A sensitive search strategy was deployed for the qualitative systematic review to identify all the studies relevant to our research question. We oriented ourselves on the PICo (population, phenomenon of interest, and context) scheme supplemented by design. The search terms used in this study are listed in Textbox 1. These were adjusted slightly to fit the different search systems, such as using Medical Subject Heading terms in MEDLINE or subject headings in CINAHL. The search was conducted using the following databases: MEDLINE, CINAHL, PsycINFO, SocINDEX, GeroLit, and Bibnet. In addition, a hand search was performed in Google Scholar, Social Science Open Access Repository (SSOAR), and in specific journals: *Technology and Health Care*, *Neurorehabilitation*, *Technology and Disability*, *Journal of Ambient Intelligence and Smart Environments*, *Telemedicine and eHealth*, *Telemedicine and Telecare*, *Journal of Applied Gerontology*, *Archives of Geriatrics and Gerontology*, and the German journals—*Pflegezeitschrift*, *Pflege*, and *Pflege & Gesellschaft*. Subsequently, a literature search of the reference lists of relevant studies was conducted.

**Search strategy.**

**Category and search terms**

**Populations**

(patients OR resident* OR “in need of care” OR “care needs” OR impaired OR disability OR disabled OR geriatric* OR elderly OR old OR “old age” OR “chronic disease” OR “chronic* ill” OR “chronic condition” OR “long-term condition” OR sick OR ill OR illness OR disease OR outpatients OR “next of kin” OR relative OR relatives OR family OR “family member*”)

**Phenomenon of interest**

AND (gaming OR exergaming OR app OR smartphone OR computer OR “mobile phone” OR tablet OR “health monitoring” OR telemedicine OR “tele-monitoring” OR telemonitoring OR “tele-homecare” OR telehomecare OR “tele-health” OR “telehealth” OR “tele-care” OR telecare OR “tele-nursing” OR telenursing OR telemetry OR “tele-communication” OR telecommunication OR tracking OR robotic OR “web based” OR “web-based” OR “health informatic” OR AAL OR “ambient assisted living” OR digital divide OR digital* OR virtual OR internet)

**Context**

AND (home OR house OR flat OR community OR “flat share” OR “flat-share” OR “assisted living” OR “assisted-living”)

**Design**

AND (qualitative OR “qualitative research” OR “qualitative studies” OR “mixed-methods” OR “mixed methods” OR ethno* OR hermeneutic* OR constructiv* OR constructionis* OR phenomeno* OR “focus group*” OR narration OR observation* OR interview* OR experience* OR “grounded theory” OR “image interpretation”)

The eligibility criteria for the literature search and the study selection process are presented in Table 1. Subsequently, the term *older adults* is used to refer to individuals of older age with chronic illness or dependency on care. We defined *old age* broadly to take into account the individuality of aging trajectories, its social construction, and other influences, such as biological, psychological, and social dimensions [17,18]. We focused on older adults from Western industrialized regions as they are considered similar in terms of cultural norms and values, as well as sociopsychological aging [18]. Studies that involved family members were also included. At the same time, the inclusion of family members in studies was not necessarily required. In this study, the term *family members* will be applied irrespective of eventual involvement in informal caregiving. The term family refers to close people whom the older person includes in this group, regardless of an existing family relationship [19]. We also included additional criteria for study quality. The development of digital technologies is advancing as fast as users’ attitudes and interactions with technologies are changing. Therefore, we limited the inclusion of studies from 2000 to 2021 to focus on current technologies and the experiences of the generation that will become older adults in the near future. We have considered studies in the languages that the authors speak.

**Table 1:** Inclusion and exclusion criteria of the literature research and the study selection process.

Selection criteria	Inclusion criteria	Exclusion criteria
Population	Older adults or synonymous expressions with chronic illness and dependency on care aged ≥50 years Family members From Europe, North America, or Australia	Children, adults aged <50 years Health care professionals Physicians From South America or Asia
Setting	Rented or purchased homes of older adults Outpatient Assisted living facilities	Homelessness Home of a family member Inpatient Nursing home
Digital technology	Internet- or sensor-based digital technology Use at home in daily life to overcome challenges in aging, illness, and care needs	Technology under development Technology for diagnosis purposes Tests in laboratory situations
Experiences	Older adults using digital technology related to aging in place Family members who use digital technology with the goal of supporting older adults to age in place	Experiences stated by health care professionals Exclusive focus on technology without relation to user experience Exclusive focus on usability and acceptance or nonacceptance
Study designs	Qualitative design Mixed methods design with a separate qualitative part	Quantitative design Discussion papers Reviews Monographs and book chapters Study protocols
Study quality	Clear separation of perspectives Sufficient quality score (≥7) in the CASP^a^ checklist	No clear separation of perspectives Insufficient quality score (≤7) in the CASP checklist
Year of publication	Between 2000 and March 2021	Before 2000 and after March 2021
Language	English, German, or Spanish	All other languages

^a^CASP: Critical Appraisal Skills Program.

### Appraising and Classifying the Findings

A systematic literature search was performed in March 2019, with an update made in March 2021. All studies were imported into the reference management system. A total of 2 researchers independently screened titles and abstracts for eligibility and examined the full texts of the studies that appeared to meet the inclusion criteria, as far as this was evident from the title and abstract. Discrepancies were discussed with a third researcher. The remaining 26 studies were appraised critically by 2 researchers using checklists for qualitative research of the CASP (Critical Appraisal Skills Program) [20]. This CASP checklist enables the systematic appraisal of qualitative studies to identify strengths and weaknesses [20]. The tool appraises the quality of the studies but not the quality of the appraisal itself. Studies were excluded if they had <7 “yes” points in the 10-point questionnaire. Consequently, 8 studies were excluded because of insufficient quality. An overview of the critical appraisals of the included studies is provided in the Multimedia Appendix 1.

Sandelowski and Barroso [15] recommended classifying the findings with regard to the methods that were used and the manner in which the data were interpreted. Hence, the included studies were classified as one of the following: thematic surveys, conceptual or thematic descriptions, or interpretive explanations.

### Conducting a Meta-summary

Two researchers independently extracted the following data from the included studies: authors, location, population, age of older adults, focus of interest, used methodology, and used technical devices. The studies were transferred to MAXQDA 2020 software (VERBI GmbH), which was used to support and manage the analysis process. We considered the results of the included studies to be interpretations of the collected data. Therefore, the results sections were treated as transcripts and used as data. The studies were read several times, and the results sections were analyzed inductively. Parts referring to the perspective of health care professionals were not taken into account. As a first cycle method, we used initial line-by-line coding. We posed the following question to the text: what are the positive and negative experiences of those affected and their family members with digital technology in terms of use, daily life, and their illness? This was followed by axial coding as a second cycle, which led to the first descriptive categories [21]. The meta-summary reflects the contents of the included studies.

### Developing a Meta-synthesis

Axial coding was combined with constant comparison [21]. The codes were grouped by constantly comparing similarities and differences. Both helped discover patterns and reassemble the data in categories and subcategories, which facilitated the formation of a hierarchical tree structure. The leading questions included the following: what added value and fears do affected individuals identify, which processes characterize use, and how did they make a decision? Theoretical coding was used as the third-cycle method to gain a deeper theoretical level of abstraction [21] and develop more generic categories. The main concepts emerged through this interpretive approach, and a conceptual model was derived.

### Ethics Approval

Ethics approval for this study was not necessary as it was a qualitative systematic review and meta-synthesis of published articles and did not involve data collection from participants.

## Results

### Overview

**Figure 1:**
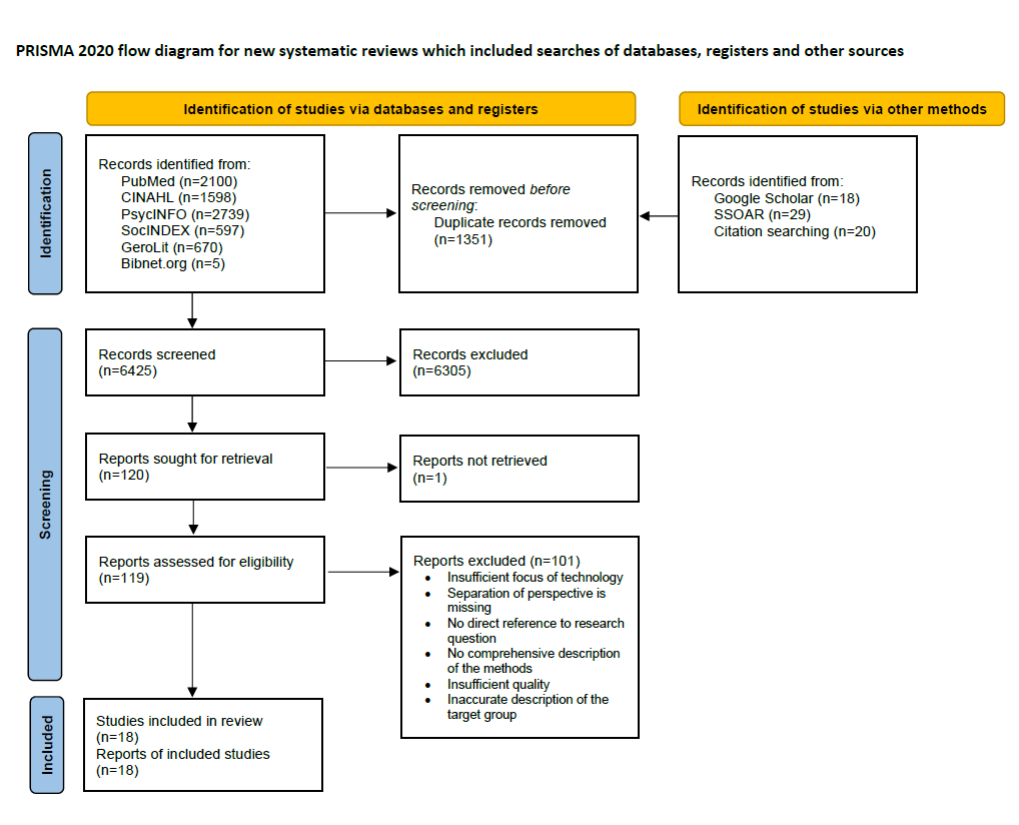
Flow diagram of the review process.

A total of 7776 studies were identified based on a systematic literature search of databases and a hand search. Furthermore, 119 studies were examined in full. A flow chart of the literature search is shown in Figure 1. Finally, 18 qualitative studies involving 220 older adults and 37 family members were included in the meta-synthesis. The study characteristics are summarized in Table 2. Older adults received care mostly in community-dwelling surroundings in 89% (16/18) and in assisted living residents in 11% (2/18) of studies. Data were collected through interviews in all the studies. Additional observations were part of 17% (3/18) of studies, and focus group discussions were part of 11% (2/18) of studies. The methodologies used were grounded theory, content analysis, thematic analysis, phenomenology, and systematic text condensation analysis. The ages of the older adults ranged from 52 to 101 years. The studies were conducted in seven different countries: Sweden, Denmark, the United Kingdom, the United States, the Netherlands, Scotland, and Australia. The technology included telehealth applications, telecommunication technology, ambient assisted living technology, exergame platforms, and wearables.

**Table 2:** Characteristics of included studies.

Authors	Location	Population	Age (years) of older adults	Focus of interest	Methodology	Technical device
Chao et al [22]	United States	15 assisted living residents with functional deterioration	78-92	Experiences of assisted living residents with facilitators and barriers with respect to exergames in relation to cognitive, physical, and psychological effects	Content analysis	The Wii-Exergaming intervention includes gaming activities for improving their functional impairment.
Chen et al [23]	United States	13 people with stroke	52-86	Experiences of benefits and barriers when using a telerehabilitation system	Thematic analysis	The telerehabilitation system comprises treatment sessions in the form of daily guided rehabilitation games, exercises, and stroke education.
Emme et al [24]	Denmark	9 people with COPD^a^	Mean 67.6	Coping with physical, emotional, and social problems of individuals with acute exacerbation of COPD before, during, and after virtual admission	Grounded theory	Virtual admission comprises virtual scheduled ward rounds via videoconferencing systems and medical equipment as monitoring devices.
Göransson et al [25]	Sweden	17 people with different comorbid conditions	70-101	Experiences with an app for supporting older people’s health and self-care	Thematic analysis	The app “Interaktor” is used to report health problems and receive evidence-based self-care advice and links to relevant websites. Caregivers can access the generated information via a web interface.
Gorst et al [26]	England	8 people with COPD; 5 family members	58-84	Beliefs and perceptions of individuals with COPD in using home telehealth	Phenomenology	Peripheral telemedicine devices were used to monitor one’s vital signs such as blood pressure, oxygen level, pulse, temperature, and weight.
Killin et al [27]	Scotland	10 individuals with Alzheimer disease or dementia; 10 family caregivers	66-81	Experiences of families with a diagnosis of dementia using a digital support platform	Thematic analysis	It involved an internet-based support platform that combines three different technologies: Living It Up, Jointly, and ClickGo. It enables families to receive comprehensive knowledge about dementia.
Klompstra et al [28]	Sweden	14 people with chronic cardiac diseases	56-81	Preferences, attitudes, use, and abilities of individuals with heart failure when using an exergame platform	Content analysis	The exergame platform (Nintendo Wii) enables to play games such as basketball, boxing, bowling, tennis, and golf. Using remote control, patients learn to play these sports in a way similar to real life.
LaFramboise et al [29]	United States	13 people with heart failure	Mean 68	Experiences about ease of use, efficacy, and difficulties of individuals with heart failure using a home communication device	Content analysis	“Health Buddy” is a telehealth device that allows people to record the status of their heart failure symptoms and receive information about their health state.
Lie et al [30]	England	21 people with chronic age-related health conditions; 11 family members	≥65	Experiences of older people with a home monitoring system focused on the acceptability, use, design, and trust of the system	Thematic analysis	The “SHel” home monitoring system comprises a home hub that communicates with wireless passive infrared sensors to monitor people’s activities.
Lind et al [31]	Sweden	12 individuals receiving palliative home care; 4 spouses	58-79	Experiences of individuals receiving palliative home care with a pain diary and digital pen and understanding their perception of pain control	Cross-case content analysis	The “Digital pen” technology comprises a pen and ordinary paper with a printed close-to-invisible pattern read by a camera inside the digital pen. This tool is intended for follow-up pain treatment, with assessment and documentation of their pain.
Lind and Karlsson [32]	Sweden	14 people with heart failure; 2 spouses	Mean 84	Experiences of older individuals with heart failure and spouses using a digital health diary and a digital pen technology	Content analysis	A telemedicine diary and digital pen are used for daily assessment. With this telehealth equipment, health care professionals can monitor patients’ daily reports via a mobile internet connection.
Mathar et al [33]	Denmark	6 people with COPD	67-83	Experiences and preferences of individuals with COPD using tele–video consultations after discharge from hospital	Systematic text condensation method	The tele–video consultations comprise eight 30-minute live investigations. During these sessions, medical professionals make observations to examine their general well-being and give advice on their medication.
Olsson et al [34]	Sweden	11 people with mild dementia	62-72	Perceptions of a passive positioning alarm for people with dementia	Content analysis	A passive positioning alarm is a GPS that includes a transmitter and a receiver. In addition, communication is possible through a loudspeaker function and getting help by pushing a button.
Selman et al [35]	England	12 people with heart failure and COPD	Mean 71.2	Experiences with a yoga intervention in relation to acceptability, appropriateness, and potential active ingredients for people with COPD and heart failure	Content analysis	The multipoint videoconferencing system enables 1-hour teleyoga live stream classes at home to receive personal instruction from the coach.
Shulver et al [36]	Australia	13 individuals undergoing rehabilitation; 3 spouses	60-92	Experiences of community-dwelling participants with a home-based telerehabilitation program and its acceptability	Thematic analysis	The home-based telerehabilitation program involves off-the-shelf technologies with tracking of activity data from the FitBit and having video calls via iPads with the therapists.
Smaerup et al [37]	Denmark	7 people with vestibular dysfunction	67-86	Experiences with computer-assisted home training for vestibular rehabilitation with a focus on self-efficacy, motivation, and acceptance	Meaning Interpretation Analyses (phenomenology)	The computer-assisted rehabilitation program “Move it to improve it (Mitii)” is personalized for patients exercising at home. Therapists can adapt the program to individual needs.
Starkhammar and Nygard [38]	Sweden	7 people with memory impairment; 7 relatives	66-87	Experiences from older adults and their families with a timer device for the stove	Grounded theory	The timer device was installed in existing electric stoves, which protects the user from fire hazards and allows the user to receive a warning signal if they forget to turn off the stove.
van Hoof et al1 [39]	The Netherlands	18 community-dwelling people with different chronic illnesses	63-87	Experiences, needs, and motives of individuals aging in place with new ambient intelligence technologies	Content analysis	The unattached autonomous surveillance system comprises the following various functions: mobility monitoring, voice output, fire detection, and wander detection and prevention.

^a^COPD: chronic obstructive pulmonary disease.

**Figure 2:**
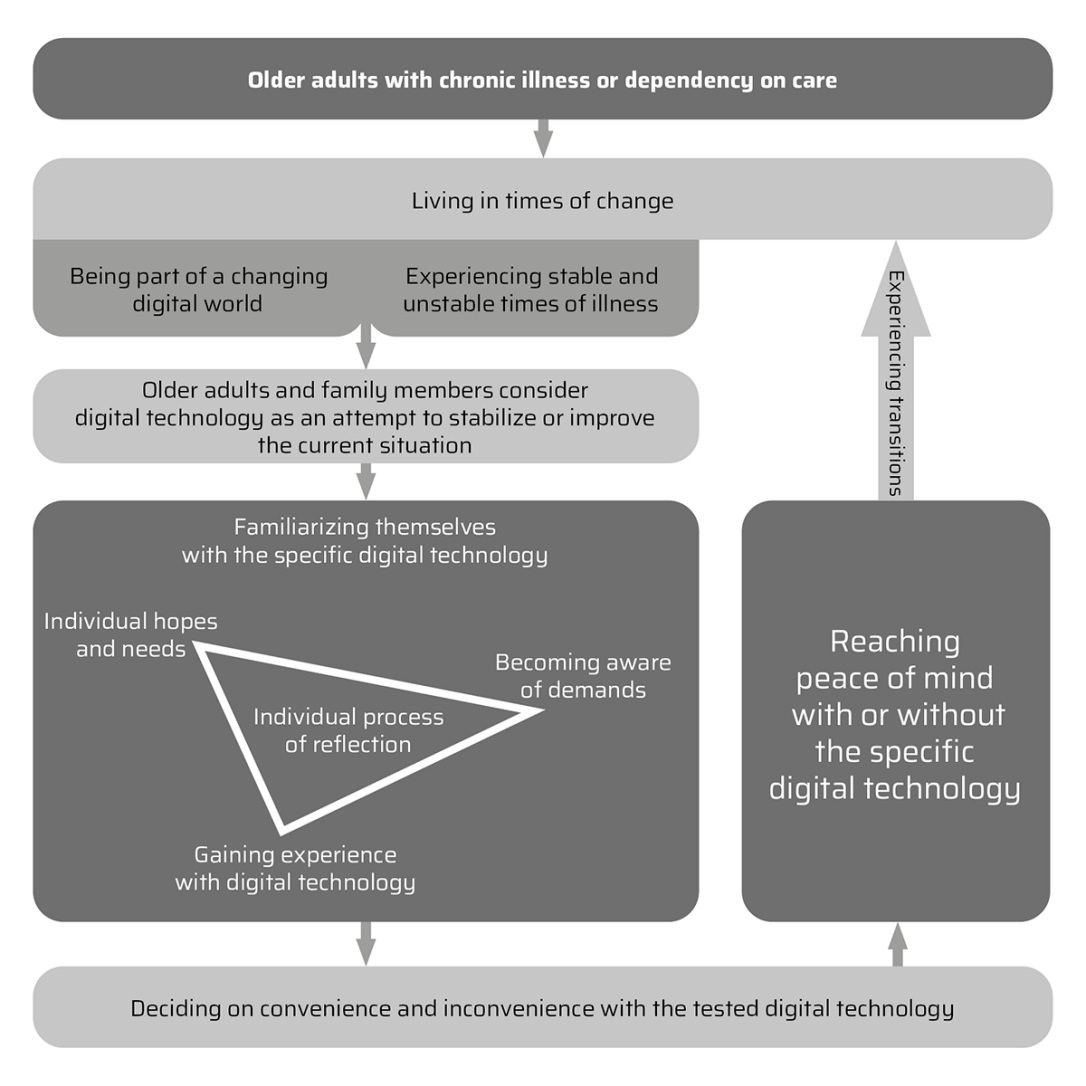
Conceptual model of older adults’ and family members’ experiences with digital technology.

To answer the research question, a conceptual model was synthesized based on an inductive interpretive analysis process for the included studies. The conceptual model derived from this comprises 3 concepts in an ongoing process that reflect the experiences of older adults aging in place and their family members with digital technology (Figure 2). Table 3 provides an overview of the identified concepts, categories, and subcategories.

**Table 3:** Concepts, categories and subcategories (excerpts from data analysis).

Concepts and categories			Subcategories
**Living in times of change**			
	Experiencing stable and unstable times of illness	Having substantial experience with the illness Being confronted with worries, insecurities, and insufficient coping strategies	
	Being part of a changing digital world	Seeking new ways to age in place Considering digital technology to stabilize or improve the current situation	
**Familiarizing oneself with digital technology**			
	Having initial hopes and needs	Wishing to age in place Not wanting to rely on others Wanting to improve health condition Wanting to challenge themselves	
	Becoming aware of demands and further needs	Noticing further needs Having to adapt	
	Gaining experience with digital technology	Overcoming initial difficulties Gaining confidence in the use Recognizing advantages and disadvantages	
**Individual process of reflection to decide on convenience and inconvenience**			
	Weighing hopes, needs, demands, and experiences	Becoming aware of changes Reflecting (un)consciously Noticing what is important for oneself	
	Deciding on convenience and inconvenience	Being (not) ready to make concessions Considering digital technology for future use Gaining peace of mind	

### First Concept: Living in Times of Change

#### Overview

The conceptual model shows that older adults live in times of change. As they experience stable and unstable times of illness, they have substantial experiences with the illness and are confronted with worries, insecurities, and insufficient coping strategies. At the same time, they are part of a changing digital world. They seek new ways of managing illness or care needs, which promote their wish for aging in place, and consider digital technology as a possible solution.

#### Experiencing Stable and Unstable Times of Illness

Chronic diseases often involve hospitalization or contact with health services and rehabilitation schemes. Individuals’ expertise with their illness sometimes comprised years of experience, in which they gained knowledge, learned coping strategies, and tested them. When older adults were confronted with illness-related problems, they applied previous coping strategies that they were confident about and had positive experiences with [24,33].

However, individuals with chronic illnesses or dependence on care experience worries, insecurities, and insufficient coping strategies. They also experience loss of control when their coping strategies are insufficient. Thus, dependency on family members and health care professionals could arise [24,33]. Emme et al [24] called this a struggle to be in control of life. Affected individuals worried about whether they could age in place in the future. They anticipated a worsening of their physical condition and feared being lonely. When they noticed insufficient coping strategies, such as during the worsening of illnesses, they experienced feeling powerless and vulnerable [24,33].

#### Being Part of a Changing Digital World

Older adults and their family members have the fact that they are part of a changing digital world in common. Older adults had different extents of experience with, knowledge about, and attitudes toward digital technology. Their attitudes ranged from being skeptical to being indifferent to being motivated to learn something new. Although some were confident about their ability to learn, others doubted their ability to manage it [22,32,36,37,39]. Some had already used technology before joining the respective studies, mainly to promote feeling safe (eg, burglary alarms or emergency response systems) [30,39].

Nevertheless, older adults seek new ways to age in place because of their chronic illness or dependency on care. They considered digital technology as possibly helpful, some with the intent of just trying, and others with clear aims to improve their situations. Older adults hoped to age in place, whereas family members were glad to have digital technology as a fail-safe system and saw it as a prerequisite to enable aging in place. Family members felt a certain responsibility during the acquisition process [25,27,30,33,38,39]:

Technology was seen as a way to support the wish to age-in-place and, therefore, embraced, accepted or tolerated as a support tool [39].

### Second Concept: Familiarizing Oneself With Digital Technology

#### Overview

Older adults already have individual hopes and needs when they learn digital technology and become aware of their specific demands while using it. During the process of familiarizing themselves with the respective digital technology, older adults and their family members continuously meet challenges and notice advantages and disadvantages.

#### Having Initial Hopes and Needs

Older adults’ intrinsic motivation to test digital technology varied. Motivations differed from not wanting to rely on family members only or to be a burden, hoping for social contact, preventing harm, maintaining or regaining autonomy, and controlling or improving their health condition with the help of digital technology. Some even saw digital technology as a type of last resort [22,24,30,35-37,39]. Others wished that digital technology would take away the decision whether to alarm family members and would support being acknowledged in the illness situation by health care professionals [24,30]. Again, others wanted to challenge themselves with something new or decrease the generation gap between themselves and the younger generation by keeping up with the times, so that they could join conversations as they did not want to be old-fashioned [22,25,28,36].

Well, if the kids can do it, I can do it [36].

Older adults showed self-efficacy toward technology as they believed in their ability to manage it [32,37].

#### Becoming Aware of Demands and Further Needs

Older adults had individual hopes and needs regarding the use of digital technology. These were complemented by the awareness of further needs and the concretization of demands while gaining experience with their use. An example was the alterations that must be made in the usual surroundings to fit in digital technology. Although some older adults “see the technology with all its implications as a part of the home or as a part of the interior design” [39], others find the rearrangement of the furniture and the lack of flexibility in placing the device inconvenient [29,35]. Older adults “found it bothersome that placement of the Health Buddy had to be where there were both an available phone jack and electrical outlet” [29]. This resulted in older adults having limited opportunities to place the digital technology, which can lead to being annoyed by the location [23,29]. Hence, they became conscious of their demand to maintain their usual surroundings only when digital technology was built in.

Another example of becoming aware of new demands is an adaptation to daily routines. Although some older adults appreciated the flexibility in the use, for others, the development of new routines was helpful. The flexibility to use digital technology at any time during the day was appreciated if other appointments or tasks got in the way [23,28,29,32,33]. However, if the device was not intended to be used flexibly, older adults did not adhere to the recommended assessment times and did so with less frequency or at different times [31]. The establishment of new routines, when successful, supported older adults in their self-management [28,29]. Helpful routines could also be established through scheduled videoconferencing times [23,24,36]. Structured programs with planned and supervised exercises “requiring a commitment on their part motivated them to exercise regularly and adhere to an exercise program” [22]. However, committing oneself to an aim or being motivated did not seem sufficient for all older adults. Reminders from family members or the digital technology itself supported older adults in using it and adhering to their routines [23,28,29,31]. The use of a certain digital technology can be closely connected with the effort to adapt previous routines. Although some older adults appreciated the structured procedure, others required reminders to use it, whereas some appreciated flexibility.

Similarly, family members associated hopes, needs, and demands with the use of digital technology. They wanted to support older adults’ wishes regarding aging in place. At the same time, the device satisfied their need for safety as they were less worried and mentally relieved [26,31,34,39]. Family members saw digital technologies as supportive while having the need to support themselves and appreciated the ability to approach the support of the platform when required [23,27,39].

#### Gaining Experiences With Digital Technology

For older adults and their family members, the use of digital technology was a learning process that required some time. They benefited from past experiences in building acceptance with the respective digital technology and facilitating its use [24,25,27,34,37,38]. Family members wanted to be prepared to support individuals with cognitive impairments for whom it was helpful to learn through several senses. Older adults with cognitive impairments familiarized themselves with the device, for instance, by being instructed verbally by technicians or family members, by reading instructions on little memos, watching other people use it, and experimenting with it [27,38,39]. It was helpful to have a program or digital technology tailored to older adults’ needs to motivate and ensure a feeling of safety, such as modifying exercises or adapting digital technology to prevent false alarms caused by pets [22,30,35,39].

Older adults experienced difficulties in handling software and hardware [27,29,31,37]. This resulted in older adults not using parts of the technology or having difficulties in their use, for instance, because of impaired sight [31,32,39]. People with memory impairment felt challenged not to forget charging or where they placed the device [34]. For them, it was demanding to remember the handling, such as necessary actions to reset the stove timer or perform actions on a written memo. Individuals with cognitive impairments continuously required reminders [38]. Older adults tried to solve problems that arose on their own through experimentation or relied primarily on help from family members or friends. Most hesitated to contact IT support in case of hardware or software problems [27,31,32,37,38], whereas others felt reassured over the availability of technical support and expected rapid responses [23,36,37].

After having managed initial insecurities, older adults gained confidence with respect to use, irrespective of cognitive impairments or terminal illnesses. They could apply the different functions and could even become enthusiastic about being able to use them, increasing skills or the benefits that they noticed [22,23,25,27,28,31,33,36-38]. They enjoyed the digital technology, even looked forward to using it, and noticed an increasing sense of well-being [22,23,25,27-29,34-36,39]. With increasing use, older adults reported improvements in their mental and physical conditions. They noticed that the potentially negative outcomes were diminished. Depending on the tested digital technology, they were more attentive and more relaxed, whereas others reported an increase in physical activity and fitness through physical exercise, for instance, by exergaming or telerehabilitation [22-24,28,29,35,36,38]. Older adults were more easily aware of symptoms and deteriorations with the help of digital technology. They were able to cope with different situations on their own, with medication at hand, or with health care professionals via telehealth. Depending on the tested technology, they were virtually admitted if needed or contacted by health care professionals about relevant issues; thereby, older adults could be cared for at home. Not needing to go somewhere for an appointment and being able to return to everyday life as soon as possible was appreciated and time saving [23,24,26,32,36,37].

Further positive experiences referred to not having to be in control. For example, during virtual admission, older adults were able to hand over responsibility and control to health care professionals who took over disease management [24]. People with cognitive decline were less anxious about the stove timer as they did not need to be in control as before [38].

Older adults also experienced increased, continuous, and improved contact with health care professionals [23,25,26,31,36]. They experienced social support through digital technology, although they did not have more frequent human interactions. For most, contact via devices was equal to face-to-face visits [26,29,36]. Communicating health concerns via digital technology, such as video consultation or a digital pen, gave older adults a sense of security as they felt having a direct connection to and “closer contact” with health care professionals. They felt the need to have positive relationships with health care professionals and did not perceive telehealth as a barrier [23-25,31-33,36]. Older adults wished to receive appreciation and feedback. They perceived the reporting of health concerns via digital technology as appreciation and support [25,31].

However, there were also older adults who wished for more social interaction with other affected persons or health care professionals. Some missed the contact with health care professionals to receive specific feedback or have the opportunity to ask questions. Although for some, it was only important to receive individual feedback, others valued face-to-face contact [26,33,35-37].

For some older adults, the use of the tested digital technology was repetitive or boring, concerning content and exercises that had limited options and were seen as monotonous [28,29,37]. Older adults were annoyed by problems or characteristics of the technical infrastructure or device, such as unreliable internet connections, false alarms, humming, and lighting, as well as negative auditory and visual feedback in exergaming [22,29,35,38,39]. They reported not having time to use the tested device; seeing it as an additional obligation; and prioritizing other things instead, such as holidays. Hence, over the period of use, some older adults became less adherent [27-29,33].

A central wish of older adults was to be self-determined in their decision to use digital technology. At the same time, they and their family members experienced uncertainty about the “right” timing. The discussion around timing is especially evident in individuals with cognitive decline. That is why individuals with cognitive impairments, having tried the device, suggested introducing the device early to promote self-determination of individual persons themselves, so that they can test it, are familiar with the device, and regard it as an aid [34,38].

### Third Concept: Individual Process of Reflection

#### Overview

This concept describes the process of reflection in which older adults and their family members were engaged in deciding on the convenience and inconvenience of the digital technology tested. They consciously or unconsciously reflected on their individual hopes, needs, demands, and experiences. Hence, they decided on the convenience or inconvenience of the tested digital technology. Irrespective of their decision, participants achieved peace of mind, some with digital technology and others without.

#### Weighing Hopes, Needs, Demands, and Experiences

##### Overview

Reflecting, in this context, is not a set process of concretely identifying hopes, needs, and demands and weighing pros and cons but a noting of what is important to oneself, incorporating hopes, needs, and demands. A participant highlighted feeling safe because of telehealth:

Like I say it’s reassuring, it’s like having another person with you even though it’s a machine. I think that’s the thing about it. It’s because I live on my own, isn’t it? I know my son is only a phone call away but I feel more reassured now that’s in [26].

**Figure 3:**
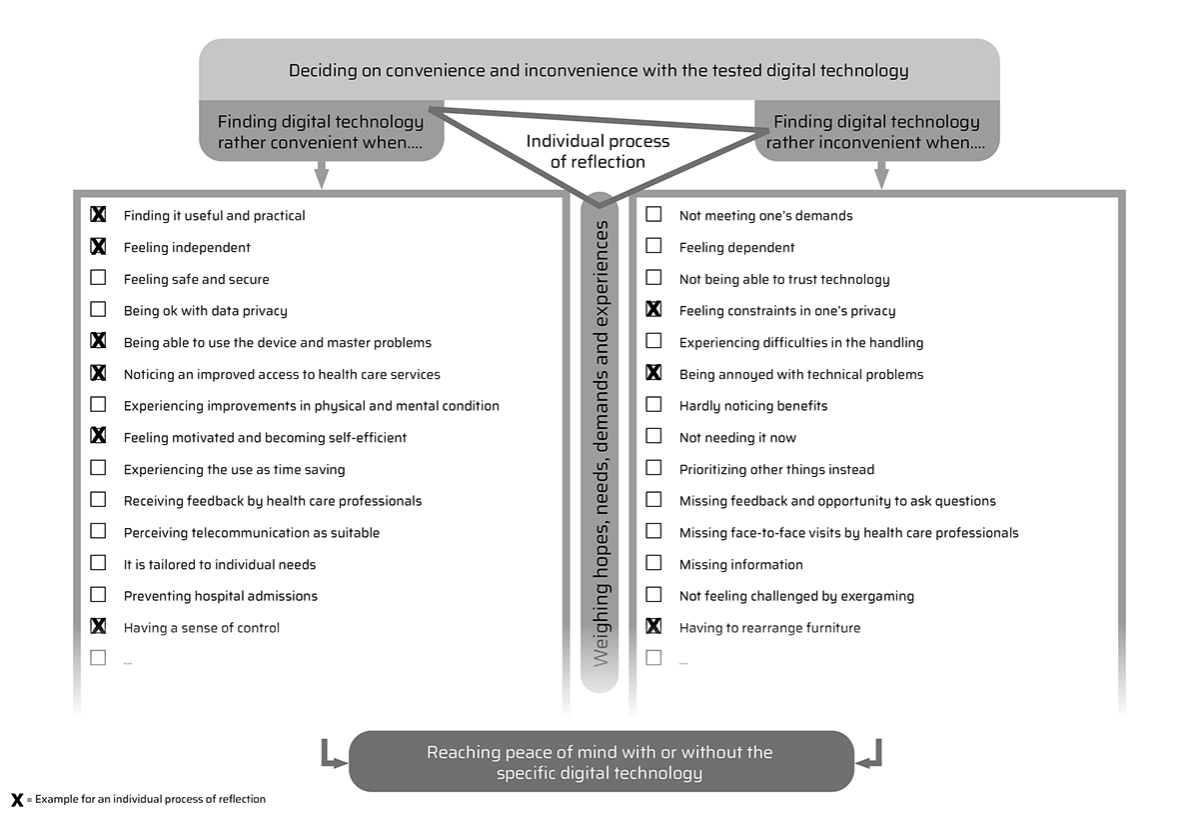
Aspects considered in the process of reflection.

Therefore, reflection in decision-making is more deliberate for one person than for another. The analysis revealed aspects based on the hopes, needs, demands, and experiences of older adults and their family members, which they considered in their individual reflection process. The results are shown in Figure 3. For example, older adults found digital technology convenient when they noticed its usefulness and practicability. They then appreciated it as an alternative to conventional offers such as doing teleyoga alone instead of exposing themselves to a group or doing exergaming at home when there was bad weather [28-30,32,35,36]. By contrast, when older adults and their family members found digital technology inconvenient, they usually noticed that it did not meet their demands. They then did not see the need to use it any further as they hardly noticed benefits in coping, fitness, self-managing behavior, or care participation [22,24,26,28,30,32,33]. For example, individuals with cognitive impairment reacted with resistance as the digital support platform confronted them with their diagnosis or one felt it was more relevant for family members [27]. A family member explained that he had a close relationship with his father and frequent face-to-face contact where digital technology was not needed [30]. Therefore, every person has different aspects that are important to them personally and that are considered in the reflection process. Hence, the aspects listed in Figure 3 need not apply to every person. Finally, reflection led older adults to decide whether they found the digital technology more convenient or inconvenient.

The reflection process is explained below in terms of 3 key themes within the hopes, needs, and demands identified: wishing to be safe, thriving for independence, and wanting to be in control. In doing so, it becomes clear what this process of reflection by older adults and their family members can look like. In the example, they weighed whether the safety, independence, or control they gained from the digital technology they tested were in relation to the individual concessions they would have to make, such as arising dependency and restricted privacy.

##### Wishing to Be Safe

Feeling safe at home is a prerequisite for aging in place. Being able to stay at home supported by digital technology provides a feeling of safety, confidence, and comfort [25,31,37,39]. The feeling of safety developed through the 24/7 availability and continuity of digital technology—knowing that health care professionals would be alerted by the system or the older adult, if required [24-26,31,33]. Even family members could increase their feelings of security through tested digital technology [26,34,38,39].

##### Thriving for Independence

Digital technology also facilitates self-determination and promotes independence, such as being virtually admitted to the hospital while being able to stay in one’s usual surroundings [24,39]. Individuals with cognitive impairments noticed increasing freedom and independence through the use of a passive positioning system [34]. Being able to manage digital technology and becoming active in improving one’s health condition promoted this feeling of independence. They noticed feeling empowered to care for their own health, being self-sufficient, and taking over responsibility [22,25,26,28-31,35]. An interviewee stated about the tested health buddy device:

It taught you how to take care of yourself and do it on your own, because nobody else is gonna do it for you [29].

Interestingly, taking over a greater part of their own care also mediated a sense of increased security [31,32].

##### Wanting to Be in Control

Older adults gained greater knowledge about their illness and symptoms through the information provided via their digital devices. Being able to use digital devices, interpret monitoring data, and contact health care professionals whenever necessary provided older adults with a sense of control over their illness management. Some older adults experience insecurities over when to approach health care professionals when experiencing an exacerbation of chronic obstructive pulmonary disease. When they are unsure of their health status, they can refer to actual and past telehealth data to confirm their symptoms [23-26,29,31-33,35,37]. An interviewee who did tele yoga said the following:

When I started to get really short of breath and I saw those numbers bang up, and I was thinking okay, I’ve got to slow down. So I went into the yoga breathing [...] and just slowed it down. And the nurses were amazed that I could do it, yeah...Because most adults hyperventilate, because they get anxious [35].

##### Example of the Process of Weighing

The positive experiences made in relation to the 3 explicated themes must be weighed against further hopes, needs, and demands that older adults and family members have and the experiences they face. They must reflect on whether they want to make concessions or not.

Lie et al [30] “found inherent tensions in maintaining both safety and privacy at the same time, as in some cases, a certain amount of privacy had to be given up for the sake of safety.” Privacy is seen as important and closely connected to being in control, as well as independent. Although some valued control over their privacy by exerting data sovereignty, others had no concerns regarding privacy and confidentiality. They did not feel invaded in their privacy or did not see telehealth as intrusive surveillance [26,30,34-36,39]. Older adults have a sense of losing independence by being watched [30,39]. They describe monitoring as “invasion of privacy” [30], or “big brother is watching you” [34]. Some participants felt restricted in their autonomy as they did not want to be watched, either via digital technology or by their families [30]. When using digital technology, older adults must trust the confidentiality of their own data. Being able to trust promotes feeling safe and, thus, acceptance of the system [30,36,38]. An interviewee (daughter of an older adult) explained it as follows:

You don’t want to go to a care or nursing home, and then you have to make some concessions of course. [...] it is not like Big Brother, it is just a sort of assistive device to stay here for longer [39].

Modern technologies such as medical equipment can also provoke a certain dependence while simultaneously providing independence [24,39]. An interviewee reflected on seeing the health care professional as responsible for monitoring his health before he used digital technology. This placed him in a dependent position. With the use of digital technology, he was able to self-manage his health [26]. Dependency also became clear through the reluctance of participants to return the device after the study [24,29,39]. For others, dependency became evident through the number of digital technologies in their households [39]. This dependency on assistive devices, in return, creates a feeling of security. Telehealth can mediate “a positive experience of surveillance” [26]. Older adults felt well cared for and watched over because they were connected to health care professionals via digital technology and interacted with the digital technology on a daily basis [25,26,29,31-33,39]. An interviewee explained it as follows:

sort of a lifeline; you know that it’s going somewhere else [...] Knowing somebody is at the end of the line, that’s important [26].

Older adults noted that using digital technology created additional obligations that were unrelated to the independence they gained,

The security that the equipment can give the patients must thus be weighed against the obligations it creates [33].

Obligations refer to solely using it or being at home for scheduled remote sessions at the agreed time [29,33]. Older adults and family members weighed their experiences with their inherent hopes, needs, and demands to determine whether they wanted to make these concessions.

#### Deciding on Convenience and Inconvenience

For older adults, achieving peace of mind means that in the process of weighing needs, demands, and experiences, some aspects receive higher priority from the individual and lead to decisions on convenience and inconvenience. Consequently, the necessary individual concessions are accepted as they do not play a leading role. The individually higher-ranked aspects lead to peace of mind. Decisions on convenience and inconvenience and gaining peace of mind also result in further actions, such as wanting to maintain digital technology, buying it, organizing one’s situation without digital technology, and maintaining health-promoting measures.

The larger group of participants gained peace of mind *with* the use of their tested digital technology. They found it convenient and were ready to make certain concessions, such as being dependent on the technology and having to give up a certain amount of privacy. Two participants were not keen on the system’s false alarms. They arranged with them and kept them out of health concerns [39]. Feeling safe was identified as the aspect receiving individually higher priority, mainly contributing to peace of mind for older adults and family members. Feeling safe not only referred to accessibility to health care professionals but also resulted from the feeling of being watched over by health care professionals. Being able to check the measurements of telehealth devices themselves, and interpret them, also contributed to feeling reassured [25,26,32,33,39]. Older adults “believe that telehealth had given them peace of mind regarding their health” [26]. An interviewee said, “I can reach them [the caregivers] easier and that means a feeling of greater peace for me, which is the main thing” [31]. Reaching peace of mind also referred to family members who felt relieved:

it’s given him peace of mind completely too. [...] It’s made us both have a life really without worrying [26].

Older adults promoted different actions as a consequence of peace of mind. Some older adults were so convinced about the device that they considered buying it themselves [24,26,30,36]. For some, maintaining trust in their home “could sometimes result in more devices being installed for peace of mind” [30]. For them, study participation resulted in wanting to continue monitoring one’s own health [24,26]. They also started recommending digital technology to others when they were convinced of its functionality [22,34]. For others, testing digital technology during the respective study was a starting point in the change in behavior and coping strategies. Hence, they were motivated by the use of devices to continue with health-promoting measures, even without the device. Older adults reported being able to maintain established helpful routines, even without digital technology, such as continuing pain assessment with paper and pencil and doing exercises they learned [23,29,31,35].

Older adults who gained peace of mind *without* digital technology were not convinced of the convenience the specific digital technology gave them. They had to compromise in the form of having no digital technology. For them, having weighed hopes, needs, demands, and experiences resulted in the decision that for the moment, they were better off without digital technology. For some, it was even a relief to return to digital technology [29,30,39]. A son explained the following:

I don’t think it will necessarily erm help us [...] we are seeing each other frequently face to face and we still live quite close to each other, so it would tend to be if we thought there was a problem we would call round [30].

For other users and family members, the experience led to the insight of not needing it as still being autonomous or not having these special needs at the moment but considering it in the future, whereas others suggested that the tested devices were more convenient for users that were far more ill [27,30,33,34].

Hence, peace of mind is not a continuous state; however, with experiencing transitions in the illness trajectories, the process of considering a new digital technology or the same device at a later time can restart.

## Discussion

### Principal Findings

The conceptual model shows that older adults with chronic illness or dependency on care live in times of change. They experience stable and unstable times of illness and are part of a changing digital world. Hence, older adults and family members consider digital technology as an attempt to stabilize or improve their current situation. While familiarizing themselves with specific digital technologies, they are in an individual process of reflection. They weigh their hopes, needs, demands, and experiences to come to a decision about whether they find digital technology convenient or inconvenient. Independent of their decision, they achieve peace of mind, either with or without digital technology. The whole process can restart as older adults experience transitions in their illness trajectories and may consider a different technology or the same one later. In this section, we focus on three main aspects: first, thriving for independence while having to arrange with dependency; second, family members’ process of adaptation; and third, health care professionals’ relationships with older adults via telehealth.

### Need for a Technical Device Tailored to the Individual Situation

The qualitative systematic review and meta-synthesis revealed that older adults thrive for independence. This was 1 of the 3 main themes that hope, need, and demand were ranked around. Digital technology can necessitate concessions as one experiences dependence on digital technology or being watched. Other studies have similarly stated this dualism. Holmberg et al [40] and Barken [41] highlighted how older adults strive for autonomy and independence while having to accept home care. Barken [41] explained how older adults maintain an independent identity by taking part in their own care. However, the independent self can be limited when older adults experience insufficient support and are unable to care for themselves as they wish. Moreover, Holmberg et al [40] identified that obtaining care implied accepting certain inconveniences to be treated in their ordinary surroundings. Hence, older adults live in a continuous process of adaptation because of experiencing illness, dependency on care, and a changing social environment while balancing their wishes with their current life circumstances [40-42]. Although these studies referred to “classical” caring situations *without* digital technology, we found similar results *with* digital technology. Older adults can make decisions about digital devices and the concessions to be made.

Our results show that family members associate digital technology with the option to enable aging in place. When successfully implemented, they experienced relief and a satisfied need for security. However, depending on the illness or care needs of the older adults, family members face various challenges. They see themselves confronted with competing demands while trying to develop a fitting arrangement [43]. Family members experience transitions with their ill relatives by striving for normalcy, as indicated by studies in palliative care [44,45]. While trying to maintain normalcy, they have to adapt to new life circumstances by balancing their old and new life situations, taking into account their own needs and demands [45,46]. From our results, family members are also confronted with a changing process in arranging with the tested technology. It remains unclear how the decision on convenience and inconvenience between family members and older adults is made. This could be further researched to optimally support the adaptation process of family members and older adults.

The results revealed mainly positive experiences of contact with health care professionals. The results obtained from appreciating the continuous availability and direct interaction with health care professionals point toward improved communication via digital technology. This leads to a closer connection with health care professionals. These positive reports are probably attributable to being part of a study, as health care professionals take more time for older adults than in normal circumstances without being part of a study. By considering the literature on video-based technology, the experiences of telehealth were equated with digital connectedness between older adults and health care professionals and were perceived as strengthening their relationship through better communication [47,48]. However, there are also contrary results in the experiences of the health care professional–patient relationship related to telehealth. Rykkje and Hjorth [49], as well as Steindal et al [47], illustrated a loss of interpersonal dynamics and replacement of human contact by using chatbots, telephone, and video calls. This could result in a lack of trust, although trust is essential for a nurse-patient relationship [50]. Therefore, our results have to be considered in terms of the positive relationship between health care professionals and older adults. From these results, we cannot make a final statement about the relationship between health care professionals and older adults. Further research on realistic scenarios for the everyday use of digital technology is required.

### Strengths and Limitations

The strength of this qualitative systematic review and meta-synthesis is the conceptual model that was derived, which provides a thorough understanding of the experiences of older adults and their family members. A sensitive search strategy was used to identify all the relevant studies. However, a limitation is that the studies focused on the experiences gained in the study context. Another limitation related to the experiences that were analyzed using different methodological approaches in the individual studies. Most studies used content or thematic analysis; only 22% (4/18) of studies used phenomenological or grounded theory methodologies. It should also be taken into consideration that only studies from Western cultures were included. Hence, transferring the results to a different cultural context must be verified beforehand. In addition, only articles published in English, German, or Spanish were considered. Another limitation is that the review was not registered in the international PROSPERO (International Prospective Register of Systematic Reviews) database.

### Conclusions

We derived a conceptual model of experiences of older adults with chronic illnesses or dependency on care and their family members using digital technologies that they tested in their homes. The model showed older adults and family members in a reflection process of weighing hopes, needs, demands, and experiences to decide on the convenience and inconvenience of the specific digital technology. Irrespective of their decisions, they attained peace of mind. This is a continuous process; it can restart during individual illness trajectories with different digital technologies or the same ones later.

This meta-synthesis had several implications. In terms of practical implications, the conceptual model reveals the need for individual counseling of older adults with chronic illnesses or care needs and their family members. Their living conditions and illness situations must be taken into account when deciding on a digital technology. Above all, the variety of available technologies must be taken into account to select the right device for the individual. Moreover, the results indicate that older individuals and their family members need to be introduced to or even trained with specific digital technology. These devices should be provided or paid for by the respective public health insurance to prevent social inequality. Thus, the model can be useful for health care providers and health care professionals. The derived conceptual model can also be used to develop digital technology. This can be the basis for communication among different disciplines. When the disciplines of technology, usability, and health sciences collaborate, a common basis can be helpful for developing digital solutions with added value for many potential users. Finally, the conceptual model can be used for the training and education of health care professionals. They could be sensitized to transitions in the illness trajectory to realize how a specific digital technology can support the current situation or need to be changed, respectively, considering older adults’ needs and demands. In addition, the conceptual model supports the understanding of health professionals and users. When users are aware of the process, they can participate more consciously to stabilize or improve their current situation with the help of digital solutions.

As an implication for future research, digital technology should be explored in the context of everyday life. This approach can enable an analysis of older adults’ experiences in their relationship with health care professionals without being subjected to special conditions in the context of an evaluation study. Moreover, the future development of technological devices should integrate older adults into participative study designs. Further research is required on the decision-making processes of older adults and their family members.

## Appendix

Multimedia Appendix 1 Quality assessment using the Critical Appraisal Skills Program (CASP).

## Abbreviations

CASP: Critical Appraisal Skills Program
ENTREQ: Enhancing Transparency in Reporting the Synthesis of Qualitative Research
PICo: Population, Phenomenon of Interest, and Context
PROSPERO: International Prospective Register of Systematic Reviews
SSOAR: Social Science Open Access Repository
