# Supplementary material for: A Conceptual Model of Experiences With Digital Technologies in Aging in Place: Qualitative Systematic Review and Meta-synthesis
Source: JMIR Aging. 2022 Sep 9;5(3):e34872. doi: 10.2196/34872 (PMC9508672; doi:10.2196/34872)
Supplement: Multimedia Appendix 1 [file aging_v5i3e34872_app1.docx]

Mulitmedia Appendix: Quality assessment using the Critical Appraisal Skills Program (CASP)

| CASP  Study | 1. | 2. | 3. | 4. | 5. | 6. | 7. | 8. | 9. | 10. | CASP  Score /10 |
| --- | --- | --- | --- | --- | --- | --- | --- | --- | --- | --- | --- |
| Chao et al. (2016) | Y | Y | Y | Y | Y | N | Y | Y | Y | Y | 9 |
| Chen et al. (2020) | Y | Y | Y | Y | Y | N | Y | Y | Y | Y | 9 |
| Emme et al. (2014) | Y | Y | Y | Y | Y | Y | Y | N | Y | Y | 9 |
| Göransson et al. (2018) | Y | Y | Y | Y | Y | P | Y | Y | Y | Y | 9,5 |
| Gorst et al. (2016) | Y | Y | Y | Y | Y | Y | Y | Y | Y | Y | 10 |
| Killin et al. (2018) | Y | Y | Y | Y | Y | N | Y | Y | Y | Y | 9 |
| Klompstra et al. (2017) | Y | Y | Y | Y | Y | N | Y | Y | Y | Y | 9 |
| LaFramboise et al. (2009) | Y | Y | Y | Y | Y | P | Y | Y | Y | Y | 9,5 |
| Lie et al. (2016) | Y | Y | Y | Y | Y | N | Y | Y | Y | Y | 10 |
| Lind et al. (2008) | Y | Y | Y | Y | Y | Y | Y | Y | Y | Y | 10 |
| Lind & Karlsson (2014) | Y | Y | Y | Y | Y | Y | Y | P | Y | Y | 9,5 |
| Mathar et al. (2015) | Y | Y | Y | Y | Y | N | Y | Y | N | Y | 8 |
| Olsson et al. (2016) | Y | Y | Y | Y | Y | P | Y | Y | Y | Y | 9,5 |
| Selman et al. (2015) | Y | Y | Y | Y | Y | N | Y | P | Y | Y | 8,5 |
| Shulver et al. (2017) | Y | Y | Y | Y | Y | N | Y | Y | Y | Y | 9 |
| Smaerup et al. (2017) | Y | Y | Y | Y | Y | N | Y | Y | Y | Y | 9 |
| Starkhammar & Nygard (2008) | Y | Y | Y | Y | Y | N | Y | Y | Y | Y | 9 |
| van Hoof et al. (2011) | Y | Y | Y | Y | Y | N | Y | Y | Y | Y | 9 |

Key: Y = Yes; N = No; P = Partially Met

Note:

1 = Was there a clear statement of the aims of the research?

2 = Is a qualitative methodology appropriate?

3 = Was the research design appropriate to address the aims of the research?

4 = Was the recruitment strategy appropriate to the aims of the research?

5 = Was the data collected in a way that addressed the research issue?

6 = Has the relationship between researcher and participants been adequately considered?

7 = Have ethical issues been taken into consideration?

8 = Was the data analysis sufficiently rigorous?

9 = Is there a clear statement of findings?

10 = How valuable is the research?
